# Dietary Intake by Toddlers and Preschool Children: Preliminary Results from a Michigan Cohort

**DOI:** 10.3390/children10020190

**Published:** 2023-01-19

**Authors:** Natalie R. JaBaay, Nikita H. Nel, Sarah S. Comstock

**Affiliations:** Department of Food Science and Human Nutrition, Michigan State University, East Lansing, MI 48824, USA

**Keywords:** breastfeeding, infants, dietary diversity, nutritional guidelines, microbiota

## Abstract

Identifying the consumption patterns of toddlers and preschool children is critical to evaluating their potential for healthy development and future heath trajectories. The purpose of this longitudinal cohort study was to describe breastfeeding, nutritional trends, and dietary diversity in 12-to-36-month-old children in a Michigan cohort. Mothers completed surveys when their children were 12 (*n* = 44), 24 (*n* = 46) and 36 months old (*n* = 32). Mothers reported their child’s dietary intake in the past 24 h and intake of specific foods in the past year. About 95% of 12-to-24-month-old children in the study population were ever breastfed, with 70% consuming human milk at 6 months and just over 40% at 12 months. Over 90% of participants gave their child a bottle since birth, with 75% providing human milk and 69% giving formula. Consumption of juice significantly increased with age and ~55% of the 36-month-old children consumed juice. A larger proportion of children consumed soda, chocolate, and candy as they aged. Though dietary diversity numerically increased with child age, this did not reach significance. Gut microbiota composition and structure was not associated with diet diversity. This research lays the foundation for future work to determine which nutritional interventions may be most effective in this population.

## 1. Introduction

A healthy diet during early childhood is important to ensure proper growth and development as well as to promote both physical and mental health [1,2]. Previous studies have demonstrated that a nutritious diet is associated with better neurodevelopmental outcomes in children [3]. Breastfeeding and complementary feeding are among the first dietary exposures a child experiences, and both play important roles in determining later life health. As children reach toddlerhood, their interactions with food become more complex. This is because their developing motor skills enable them to feed themselves, and they can voice their food preferences as well as exert independence through dietary choices [4]. Additionally, children’s preferences regarding food are associated with their consumption patterns during infancy and toddlerhood [5]. Thus, early diet establishes important patterns and health trajectories for later life [1,2].

Currently, the dietary quality of children in the United States needs improvement, as many children are obese and rates of chronic diseases in childhood continue to increase. Over 8% of infants are at risk for overweight, and 14% of 2–5-year-olds are obese [6]. A rapid weight gain trajectory during a child’s first 2 years of life is associated with an increased risk of the child becoming overweight or obese in adulthood [7]. Poor diet quality is also linked to impaired cognitive function and diminished academic achievement in children [8]. Proper nutrition is needed to halt these poor health patterns. Establishing healthy dietary patterns in infancy through preschool age may prevent negative health effects from developing in the future and promote a higher quality of life [6]. Upon further examination, one underlying issue for establishing healthy dietary patterns may be the confusing dietary intake recommendations for infants and young children. In fact, the Dietary Guidelines of 2015–2020 did not address recommendations for Americans under 2 years of age. However, the 2020–2025 Dietary Guidelines include some nutrition recommendations for Americans from birth to 2 years. The Dietary Guidelines and the American Academy of Pediatrics recommend exclusive breastfeeding for 6 months and continuation of breastfeeding through the infant’s first birthday, or as mutually desired by both mother and infant [9,10]. Complementary foods should not be introduced until 6 months of age, but, when they are introduced, a wide variety of healthy foods and textures should be included, and any juices or sugar-sweetened beverages should be avoided [10]. As for toddlers and preschool age children, the Dietary Guidelines emphasize the need for nutrition requirements to be primarily met through a healthy dietary pattern of foods and beverages while limiting added sugars, saturated fat, and sodium [9]. According to the calculated Healthy Index score of 61 out of 100, children 2–4 years of age have an overall poor diet quality [9]. It is unknown if similar consumption trends are observed on a local level [11].

A hallmark study in dietary intake in children is the Feeding Infants and Toddlers Study (FITS) that collected telephone-administered 24 h food recalls in 2002, 2008, and 2016. FITS reported that 73% of infants in the study were introduced to complementary foods between 4 and 5.9 months of age, and infants who were currently consuming formula were more likely to be introduced to complementary foods before the 6-month recommendation [6]. Before their first birthday, about three-quarters were consuming fruits and vegetables, however, one-third were already consuming sweets and sugar-sweetened beverages [6]. Most notable, consumption of sweets and sugar-sweetened beverages increased with age [6,12]. Notable differences across the FITS studies that spanned from 2002–2016 were an increase in sodium levels, decreased consumption of grains, increased consumption of non-baby food vegetables in infancy, increased consumption of meat in infancy, and a decrease in the percent of children consuming any sweets or sugar-sweetened beverages [12].

Dietary diversity, or dietary variety, is defined as the variety of food being consumed, including the number of food groups, frequency, and consumption over time [13]. Dietary diversity can be scored using the minimum dietary diversity (MDD) score designed by the World Health Organization (WHO), which focuses on the consumption of the following eight food groups: human milk; grains, roots, and tubers; legumes and nuts; dairy products; flesh foods; eggs; vitamin A-rich fruits and vegetables; and other fruits and vegetables [14]. High dietary diversity within the first year of life is associated with a lower risk of developing food allergies over the first decade of life, as allergens are incorporated into the diet [13]. The early introduction of allergenic foods, such as cow’s milk, peanuts, cooked egg, fish, wheat, and sesame, may be effective at preventing food allergies [15]. Measuring dietary diversity is an important component in assessing childhood dietary intake.

As presented above, there are gaps in the literature regarding the dietary intake of children from birth to 3 years of age. Our study provides preliminary information regarding the nutritional and breastfeeding trends within a Michigan cohort. Further, we identify which recommendations are being followed and if dietary diversity differs according to an infant’s age. This study is unique in its prospective longitudinal design, as most data available are cross-sectional. Additionally, we provide insight into which food categories are most consumed within our population and report the differences between proximal and habitual diet recall. We hypothesized that children would fail to meet current nutritional recommendations but would perform similarly to the reference populations. This research within our cohort can inform future nutritional interventions on how to best alter dietary trends and improve nutrition for young children.

## 2. Materials and Methods

### 2.1. Study Design and Participants

**Figure 1:**
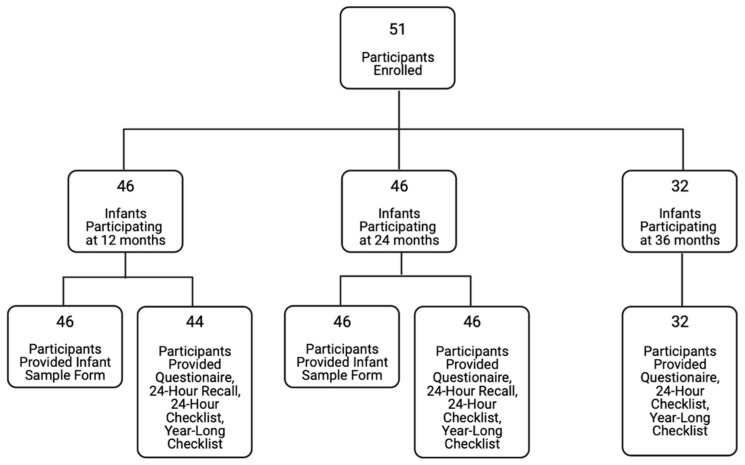
Participant flow chart.

Study participants (*n* = 51) for our cohort were convenience samples recruited from various health clinics in Lansing, Michigan and Traverse City, Michigan, as previously described [16]. Participant enrollment was open to all English-speaking, 18 years or older, pregnant women and their infants, who attended the clinics. There were no additional exclusion criteria. For this longitudinal cohort, informed consent was obtained during the medical checkup from the child’s caregiver. Parent-reported surveys were used to collect qualitative nutritional data at 12, 24, and 36 months of age (Figure 1). Participating mothers were mailed paper questionnaires once each assessment year during the specified recall periods. Participants were not provided with any nutritional information by the study but may have received such information from a healthcare provider. Questionnaires were completed in the home, returned to the lab by mail, and data was entered into a computerized database. It should be noted that intake misreporting and recall inconsistencies may affect the accuracy of reported intake and are limitations to this study. A total of 85 percent (*n* = 39) of the 24-month respondents completed both the 12- and 24-month questionnaires. A total of 91 percent (*n* = 29) of the 36-month respondents completed all 3 sets of questionnaires. At 12 and 24 months, participants completed a parent-reported 24-h diet recall, a 24-h diet checklist, and a detailed questionnaire related to human milk consumption. At 36 months, participants did not complete the 24-h diet recall, nor did they report detailed information about human milk exposure; however, the 24-h diet checklist was collected. A more general qualitative questionnaire, administered at all three timepoints, recorded dietary exposures in the past year and included additional questions about bottle feeding and complementary food introduction. This questionnaire asked mothers to recall dietary information from earlier timepoints to assess consistency in recall [17]. No dietary information was collected regarding portion size or quantity. The results from all surveys were compiled into a single database and cleaned for analysis. Microbiome samples were collected, then processed and analyzed as reported in Sugino et al. [18]. Our questionnaire tool source does not report validity or reliability [19]. This study was approved by the human subjects’ research protection program at Michigan State University.

### 2.2. Dietary Diversity

Using the information about foods consumed in the 24 h prior to the survey completion, a diet diversity score was calculated for each child at each time point using the method of the Food and Agriculture Organization of the United Nations [20]. Each reported food was categorized into one of the seven dietary diversity groups, (1) grains, roots, and tubers; (2) legumes and nuts; (3) dairy products; (4) flesh foods; (5) eggs; (6) vitamin A-rich fruits and vegetables; and (7) other fruits and vegetables. Human milk was not included as a separate group. Each participant was then given a dietary diversity score for each time point. The score corresponded to the number of categories met based on the food reported to be a part of the child’s diet. For example, if a child’s diet included items from five of the seven dietary diversity groups described above, the diet diversity score would be five. Once each participant was scored, the mean and median score for each age group was calculated.

### 2.3. Statistical Analyses

Participant demographics were compared using *t*-tests when data were normally distributed, and Wilcoxon rank sum tests when data were not normally distributed. Chi square tests were used to compare proportions. A Friedman’s test was performed to compare diet diversity by age (SAS version 9.4, SAS Institute, Cary, NC, USA).

All foods and beverages reported in 24-h and year-long recalls were sorted into a food-category checklist with specific groups. The percentage of infants from the population each year that consumed a specific food group was calculated. Once the percentage was calculated for each of the food groups for each of the three recall periods, the data were imported into R using the R Studio interface, and R was used to perform Chi-squared statistical tests using child age at survey completion as the independent variable [21,22].

## 3. Results

### 3.1. Participant Demographics

Based on responses to the Demographics Questionnaire, participant characteristics were similar across the study period. This was inclusive of maternal age, income level, marital status, living conditions, race, and infant sex (Table 1). When compared to the general 2020 Michigan population, both the race (*p* = 0.98) and education (*p* = 0.59) in the study population were similar to the general population. About 40% of the study population had an income below $50,000, which is close to the state of Michigan 2020 income median of $59,234 [23]. Compared to the general Michigan population, where male and female infants are nearly equally represented, the study population included a significantly larger proportion of male infants (74%) than female infants (26%).

### 3.2. Breastfeeding and Human Milk Alternatives

Approximately 95% of the 12-to-24-month-olds in our sample population had ever been fed human milk. This proportion is similar to the reported 2017 US average of 84.1% (*p* = 0.75) [24]. The proportion of infants currently receiving human milk decreased from 70% at 6 months of age, to 48% at 12 months of age, to 11% at 24 months of age (Table 2). There were 21 participants who answered all breastfeeding recall questions at each of the 3 survey timepoints. When those 21 participants were asked about their infant’s age when they stopped breastfeeding, 19% reported the same weaning age all three years, 52% changed the weaning age during at least one recall period, and 29% changed the weaning age each recall period. The change in recall period can most frequently be attributed to the fact that participants were still breastfeeding at the 12-month timepoint and increased the age reported at the 24-month timepoint. Notably, about one-third (33%) of participants increased the reported age of weaning in the 24-month survey to exceed 12 months. Of those infants who weaned prior to 12 months of age (*n* = 10), 30% consistently reported the weaning age over the 3 time points, while 50% changed the reported age at one recall period, and 20% changed the reported age at all three recall periods.

**Figure 2:**
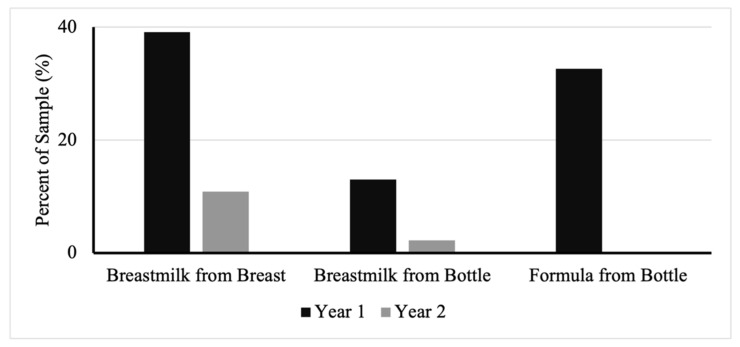
Percent of infants consuming human milk or formula in the 24 h immediately preceding survey completion.

Mothers also reported breastfeeding practices within the 24 h prior to survey completion in the 12- and 24-months-of-age questionnaires. The consumption of any human milk or formula decreased over time (Figure 2). Those in the cohort who had consumed human milk from the breast decreased from 39% at 12 months to 11% at 24 months. Those who consumed human milk from a bottle decreased from 13% to 2%. Formula consumed from a bottle in the past 24 h had the most drastic change, decreasing from 33% to 0%.

As expected, the proportion of the diet consisting of formula or other foods increased over time as the proportion of the diet consisting of human milk decreased over time (Table 2, *p* = 0.024). The percentage of children with a diet based on “100% formula or other foods” increased from 66% at 12 months to 88% at 24 months. The proportion of infants consuming any category that included human milk decreased from 12 to 24 months, with the exception that the percentage of children consuming 50% breastmilk and 50% formula remained ~2%.

Mothers also reported whether they fed their child anything from a bottle and the contents of the bottle, such as human milk, formula, water, juice, or other (Table 3). At each timepoint, over 90% of participants confirmed giving their child a bottle to drink from at some point since birth. Among those who gave their infants bottles to drink from, about 75% of participants ever gave their infant human milk in a bottle. Similarly, 69% of mothers ever gave formula in a bottle. Meanwhile, a smaller percentage of infants ever drank water (36%) or juice (18%) from a bottle.

Recall of first foods was inconsistent. Twenty-nine participants answered all food introduction questions at each survey timepoint. When asked about the types of foods introduced, out of those 29 participants, 28% reported the same foods introduced each recall period, 24% of participants had changed 1 type of food reported for introduction, and 48% reported 2 or more different first foods introduced. With respect to age of food introduction, out of the 29 participants, 48% reported the same age of food introduction for all 3 timepoints, 42% reported a different age of introduction for 1 of the timepoints, and 10% reported a different age of food introduction at each of the 3 timepoints.

### 3.3. 24-Hour Recall

Dietary consumption in the 24 h prior to survey completion was consistent for most foods from 12–36 months of age (Table 4). Food categories including meat, grains, fruits, and vegetables were the most commonly occurring food categories, and typically more than 70% of participants had consumed these groups within the past 24 h for each year that the surveys were completed. Other categories including soy, liver, fish oil, and other supplements were not commonly reported, as they had been consumed by less than 10% of participants within the past 24 h for each year that the surveys were completed. Multivitamin intake rose from 3% of the population reporting consumption in the past 24 h at 12 months, to 37% at 36 months (*p* = 0.0003). Reported consumption of foods in the categories including dairy, 100% juice, sweet food, and sweet drinks also significantly increased over the course of the 36-month study period.

### 3.4. Dietary Intake in the Past Year

Similar to the 24-h recall results, fruits, vegetables, and animal meat were the three most commonly consumed food categories in yearly diets, with each being consumed by 100% of participants for at least two out of the three years (Table 5). The fruits category was consumed by 100% of participants each year the survey was given. Fruit juice, soda, chocolate, and candy each had a statistically significant increase in reported consumption, with candy having the largest increase of those groups (*p* = 4.91 × 10^−10^). Candy was only given to 21% of infants at 12 months but reached a 91% consumption rate at 36 months. As mentioned previously, human milk consumption significantly decreased each year, and was the category with the most drastic change (*p* = 9.24 × 10^−14^). A total of 93 percent of the study population was consuming human milk at 12 months, but by 24 months only 36% of infants still included it in their yearly diet, and at 36 months only 6% of the population had consumed human milk in the past year.

In the year-long diet recall, mothers also reported the child’s consumption of different types of formula, all of which significantly decreased over time (Table 5). At 12 months of age, 68% of the cohort was consuming infant formula (including comfort/gentle infant formula, hypoallergenic infant formula, cowmilk infant formula, reflux infant formula, soy infant formula, or other infant formula). Comfort/gentle infant formula intake decreased from 52% at 12 months, to 11% at 24 months, reaching 0% at 36 months (*p*= 7.19 × 10^−8^). Cowmilk infant formula and other infant formula categories also experienced a similar significant decline, and by 36 months had less than 10% consumption by the total population. Other formula types including reflux infant formula, soy infant formula, and hypoallergenic infant formula had consistently low reported percentages and reached 0% consumption by 36 months.

### 3.5. Dietary Diversity

**Figure 3:**
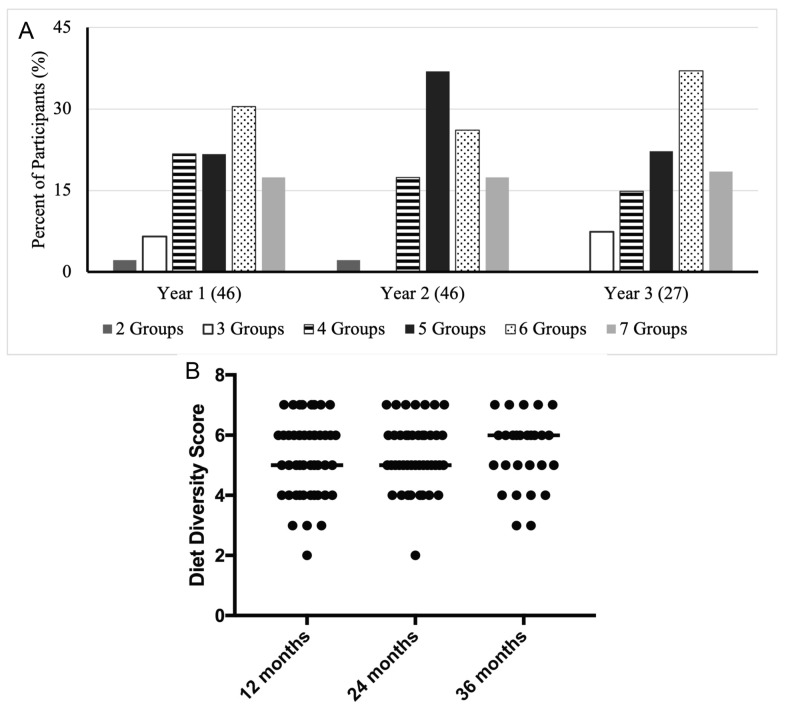
Diet diversity. Percent of participants achieving specific dietary diversity scores across the three-year study period (**A**). Dietary diversity scores at each age (**B**). Medians are indicated by solid lines.

There were no statistically significant differences in dietary diversity scores across the 12- to 36-month period (*p* = 0.827; Figure 3). These scores were calculated using the information about foods consumed in the 24 h immediately preceding survey completion. Both the 12- and 24-month-old infants attained a median dietary diversity score of 5, whereas the 36-month-old infants attained a median dietary diversity score of 6 (Figure 3B). At each of the three time points, all participants consumed at least two dietary diversity groups. Additionally, 74% to 80% of infants at each age period consumed either four, five, or six dietary diversity groups. There was some variation in the score distribution across these three groups during the 36-month period. The 12-month recall period had the largest percentage of infants with 4 groups (22%), the 24-month recall period had the largest percentage of participants with 5 groups (37%), and the 36-month recall period had the highest percentage of participants with 6 groups (37%). At each of the three timepoints, about 17–18% of participants consumed all seven dietary diversity groups.

**Figure 4:**
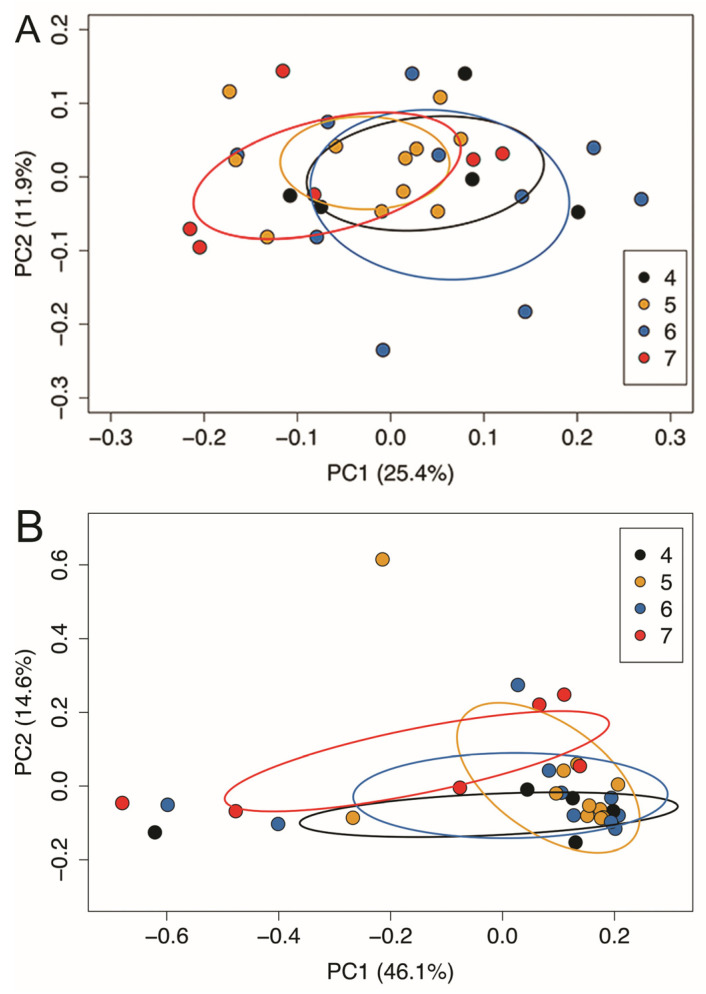
Beta-diversity of the toddler (24 months old) gut microbiota. Membership of the toddler gut microbiota was similar regardless of dietary diversity score (Sorensen metric) (**A**). Beta diversity of the toddler gut microbiota was similar regardless of dietary diversity score (Bray–Curtis metric) (**B**).

Fecal samples were also collected at the 24-month time point to determine if measures of the diversity of the toddler gut microbiota were related to the toddler dietary diversity scores. No statistically significant differences were observed in either alpha or beta diversity of the toddler gut microbiota by dietary diversity score, nor were any statistically significant associations observed when comparing those with the highest dietary diversity score (n = 8 with scores of 7) compared to the rest of the participants (Table 6). Therefore, gut microbial communities of toddlers with different dietary diversity scores were similar (Figure 4).

The seven categories of dietary diversity were also separated and analyzed for the group of participants at the 24-month time frame. It was observed that all participants had consumed both the “grains, roots, and tubers” and “dairy products” categories. The “other fruits and vegetables” and “flesh floods” categories were the third (93.5%) and fourth (84.8%) most common categories. Thus, these foods were highly likely to be consumed by those with a dietary diversity score of 4 or less. Of those with a dietary diversity score of 5, 58.8% consumed “legumes and nuts,” while in the general group of 24-month-olds, 60.9% consumed “legumes and nuts”. Furthermore, of those with a dietary diversity score of 6, 75.0% consumed “eggs”, while 54.3% of all 24-month-olds consumed “eggs”. The category, “vitamin A-rich fruits and vegetables”, was the least commonly consumed, with 43.5% of 24-month-olds consuming “vitamin A-rich fruits and vegetables” in general. All 24-month-olds with a dietary diversity score of 7 consumed “vitamin A-rich fruits and vegetables”.

## 4. Discussion

Infants in our cohort exceeded national recommendations for consuming breastmilk, the continuation of breastmilk past 6 months of age, and complementary feeding beginning at or around 6 months of age. Foods with added sugars, such as candy, chocolate, fruit juice, and sugar-sweetened beverages, were introduced in a small proportion of our population prior to the recommended age in our population, and consumption of such foods significantly increased as the children aged. Diet diversity was similar at each sampling time point. The average dietary diversity score was approximately a 5 out of 7, exemplifying that most children in our cohort consumed a varied diet consisting of fruits, vegetables, meats, grains, and dairy.

### 4.1. Breastfeeding Trends

Breastfeeding rates in our population were above both the national and state averages. The majority of children in our cohort were breastfed at some point in their life, with more than 90% breastfeeding at 12 months, compared to the Michigan average of 77.7% and the national average of 83.2% [24]. This trend of exceeding the state and national averages continues through the 6- and 12-month periods. Based on our results, our cohort exceeds current breastfeeding recommendations. This may be due to the participants’ personal characteristics. Those who agree to participate in research may also be more likely to breastfeed their children and to do so for a longer period of time.

### 4.2. Complementary Feeding

According to the 2020–2025 Dietary Guidelines, as well as recommendations previously established, complementary feeding should occur between 4 and 6 months of age [9,10]. The average age of introduction within our population was approximately 6 months, signifying that our population followed the current recommendations. In comparison, the Infant Feeding Practices Study II (IFPS II) reported that complementary feeding occurred prior to the recommended 6-month mark, with some introducing foods before the minimum recommendation of 4 months of age [25]. Regarding exclusive breastfeeding, only about 4% of infants at 6 months old were obtaining their nutrients exclusively from breastmilk according to a 7-day dietary recall among varying demographics, despite the recommendations [25]. This implies that approximately 96% of infants are already consuming complementary foods, or formula supplementation, by 6 months of age. The study population we examined met the current guidelines and recommendations for introducing solids at 6 months of age, which may be due to mothers’ awareness of and, thus, adherence to these nutrition recommendations [9,10].

### 4.3. Bottle Feeding

Current recommendations state that only human milk or formula should be fed to infants in bottles [26]. Approximately 75% of infants in our population were consuming human milk in bottles, while just under 70% consumed formula in bottles. However, around 40% were using bottles to drink water and nearly 20% were drinking juice out of bottles at the 12-month time point. Per recommendations, neither water nor juice should be fed in bottles, and juice is not to be introduced to children until 2 years of age [27,28]. The negative relationship between health outcomes and juice exposure in infancy continues as children age and consumption increases. The ease of consuming juice from bottles allows for toddlers and young children to overconsume, which may lead to energy imbalance of either over or undernutrition, diarrhea, and dental cavities throughout childhood [27]. This may be of concern for our population, as one-fifth were consuming juice in bottles and are at risk for these health outcomes. At the 12-month time period, nearly 20% of infants were also consuming “other” in bottles, where “other” is a beverage or food that does not fit in the human milk, formula, water, or juice category. The “other” category may have included cereal in bottles, as this is a common practice. A 2011 study on the infant feeding practices of children found that the young mothers in the study, who were between 19 and 22 and identified as low-income status, cited reasons for adding baby cereal to their infants’ bottles such as their child was not full after one bottle of formula alone or they were doing so in hopes of their child sleeping longer at nighttime [29]. The CDC cites evidence that cereal in bottles does not increase sleep duration and additionally increases risk of choking [9,26]. While we do not know what specific foods or beverages the “other” category constitutes in our cohort, about one-fifth of mothers in our cohort are feeding something to their children in bottles other than human milk, formula, water, or juice, which conflicts with current recommendations.

### 4.4. Consumption of Sugar-Sweetened Beverages

We hypothesized that the consumption of nutrient-poor foods and sugar-sweetened beverages (SSBs) would increase with age, and the data support this hypothesis. This was reflected not only in the 24-h recall data but also in the checklist of foods consumed in the past year. Table 4 illustrates the significant increase in the consumption of both sweet food and sweet drinks, including 100% fruit juice, each year based on 24-h recall data. Children also significantly increased their intake of soda, fruit juice, chocolate, and candy as reflected in responses to the checklist of foods consumed in the past year (Table 5). This is consistent with FITS data, as they reported a similar trend of increased consumption of sweets and sugar-sweetened beverages within the first 2 years of life [12,30]. It is important to note that added sugars should not be introduced into the child’s diet until they are 2 years old. However, even when they reach preschool age, there is limited room for added sugars in a child’s diet due to the high need for other nutrients such as protein and omega-3 fats to support proper growth and development in this stage of life [9]. The underlying causes leading to an increase in SSB consumption throughout early childhood are unclear. However, the diets of infants and children often mirror those of others in their household, specifically that of their mother or primary caregiver. Some evidence suggests that young mothers in low-income households who are overweight tend to introduce nutrient-poor foods with added sugars into their children’s diets prematurely [29,31]. This early introduction to added sugars may then cause children to prefer sweetened foods and beverages over less or naturally-sweetened options [32]. The consistent reports that toddlers and preschool children consume SSBs suggests that interventions that reduce SSB consumption are likely to decrease added sugar consumption in early childhood.

### 4.5. Fruit and Vegetable Intake

Our population achieved adequate fruit and vegetable intake. Within the first year of life, 100% of infants in our cohort consumed both fruits and vegetables, with around 96% consuming fruits in the past 24 h and 87% consuming vegetables in the past 24 h. Intake for both categories decreased at 24 and 36 months of age, which could be due to a more diverse diet, lack of interest, or introduction of sweetened foods. Compared to the FITS data on infants’ and toddlers’ fruit consumption, our population exceeded their average of about 80% of infants consuming fruit in the first year and 90% of toddlers consuming fruit in the second year [12]. The Dietary Guidelines report that about 60% of toddlers, 12–23 months of age, meet or exceed the recommended intake values for fruit consumption [9]. In early childhood, FITS data shows that approximately 77% of children ages 2–3 consumed fruit on the day of the 24-h telephone-administered recall, whereas in our population, at this age, just over 80% had consumed fruits on the day of the recall [33]. Throughout our longitudinal study, nearly all children were consuming vegetables according to the year-long checklist. On the day of the study, above 70% of our cohort had consumed vegetables, similar to the FITS study results [12,33]. According to the Dietary Guidelines, about 90% of toddlers do not meet vegetable intake recommendations, with one-half of vegetables being consumed independently and one-quarter as part of a mixed dish [9]. While we have a high percentage of children consuming vegetables, we are unsure of the quantity and variety of vegetables they are consuming, thus we cannot conclude whether the children in the study population are meeting recommendations in this category. We can conclude that these children are exceeding FITS children in fruit intake recommendations and are consuming vegetables with similar intakes to that of the children of the FITS study.

### 4.6. Supplement Usage

According to NHANES data from 2007–2014, approximately 15% of infants are given supplements within the first 6 months, 12% within the first year, and just above 20% in the second year [34]. Overall, approximately 1 in 5 infants and toddlers ages 0–24 months consume at least 1 dietary supplement [34]. Among supplements being consumed across all age groups, vitamin D supplements are the most common [34,35]. Vitamin D supplementation is recommended for infants who are either consuming human milk exclusively or a combination of human milk and infant formula soon after birth [9]. FITS data from 2016 reported an increase in dietary supplementation among infants and toddlers compared to their 2008 data and attribute this to the increased use of vitamin D supplementation to meet AAP recommendations in infancy [35]. In our population, vitamin D supplementation was not widely used, but supplementation with vitamin D decreased with age. This could be explained by infants weaning from human milk and supplementation and then relying on their diet to provide adequate amounts of vitamin D. In NHANES, multivitamins were introduced primarily within the second year of life. The reasons listed for supplement use included to maintain the child’s health, improve overall health, or to supplement the diet [34]. Our results are consistent with those from NHANES, as multivitamin supplementation in our cohort was minimal within the first year and increased in the second and third years of life. Despite the potential positive benefits of fish oil, omega-3 fatty acid, or DHA supplementation in infancy, including cognitive and visual development, as well as the possible prevention of childhood food allergies, the majority of children in our cohort did not consume these supplements in the first three years of life [36]. Supplement use within our study population was similar to recommendations and other national cohort studies [34].

### 4.7. Study Limitations

Limitations of this study include inconsistent recall of the timing of introduction to solid foods, as detailed above, as well as a small sample size. Inconsistency among participants’ answers at each appointment may skew the results and provide an inaccurate representation of the dietary quality of the children in our cohort. Many children typically consume only one meal per day within the presence of family members, which may cause some discrepancies in the foods reported [37]. Recall inconsistencies and individual metabolic differences may also cause discrepancies between measured laboratory results and described nutritional intake [38]. A smaller population size means our results may not be applicable to other populations. Our study’s generalizability is also limited due to the small number of data collection timepoints during the infant’s development. Future studies should aim to collect additional data more than once a year to better characterize fluctuating infant nutritional trends. However, our cohort shares similar demographics, including race, education level, and income level, to the general population of the state of Michigan. Additionally, our results are similar to those reported from much larger national cross-sectional studies, as described above, thus increasing our confidence in our results. When asking participants about breastfeeding, our questionnaires focused on *if* infants and children were being breastfed, not if they were *exclusively* breastfed. The exclusivity of human milk consumption would enable comparison of the study population to recommendations and the current literature. The strengths of this study include the prospective longitudinal study design, the high rate of retention, as well as the use of multiple methods for dietary intake assessment.

## 5. Conclusions

Overall, the children in this Michigan cohort are meeting basic dietary recommendations for children 1–3 years of age. Breastfeeding rates, fruit and vegetable consumption, and the avoidance of added sugars in infancy were all positive child feeding behaviors in this study population. Behaviors related to the recommendations to limit nutrient-poor foods and added sugars in early childhood were not observed in the study population. Therefore, the study provides insight regarding areas that need additional public attention and education.

## Figures and Tables

**Table 1:** Participant characteristics by child age.

Variable	12 Months (*n* = 44)	24 Months (*n* = 46)	36 Months (*n* = 32)	*p*-Value
Maternal Age at Childbirth (years)	31.4	30.9	31.3	0.799
Missing, %	4.5	4.3	6.3	-
Household Income ≥50,000 USD, %	65.0	58.1	58.1	0.993
Missing, %	9.1	6.5	3.1	-
Race, %	-	-	-	-
White	83.3	84.4	84.4	0.997
Black	11.9	11.1	12.5	
Remaining	4.8	4.3	3.1	
Married, %	83.3	80.0	81.3	0.992
Missing, %	4.5	2.2	0.0	-
Living with Partner, %	83.3	77.7	81.3	0.803
Missing, %	4.5	2.2	0.0	-
College Degree, %	68.3	65.9	71.0	0.898
Missing, %	6.8	4.3	3.1	-
Infant Sex, Female, %	27.9	26.7	22.6	0.870
Missing, %	2.3	2.2	3.1	-

**Table 2:** Breastfeeding trends by child age.

Variable	12 Months (*n* = 44)	24 Months (*n* = 46)	*p*-Value
Ever Fed Human Milk, %	93.18	95.65	0.959
Missing, %	2.27	0.00	-
At 6 months, %	56.82	69.57	0.300
At 12 months, %	36.36	47.83	0.375
At 24 months, %	-	10.87	-
Missing, %	18.18	13.04	-
Formula, Human Milk, and Food Consumption in the 24 h Immediately Preceding Survey Completion			
Variable	12 months (*n* = 46)	24 months (*n* = 46)	*p*-Value
100% Formula or Other Foods; %	60.87	88.89	0.024
80% Formula or Other Foods, 20% Human Milk; %	15.22	6.67	
80–50% Formula or Other Foods, 20–50% Human Milk; %	15.22	2.22	
50% Formula or Other Foods, 50% Human Milk; %	2.17	2.22	
20–50% Formula or Other Foods, 50–80% Human Milk; %	6.52	0.00	
20% Formula or Other Foods, 80% Human Milk; %	0.00	0.00	
100% Human Milk, %	0.00	0.00	
Missing, %	0.00	2.17	-

**Table 3:** Bottle-feeding consumption trends. Reported as percent of children consuming the food within the age group.

Variable	12 Months (*n* = 44)	24 Months (*n* = 46)	36 Months (*n* = 32)
Human Milk, %	73.2	75.0	75.9
Formula. %	68.3	69.4	75.9
Water, %	41.5	44.4	27.6
Juice, %	17.1	22.2	13.8
Other, %	17.1	27.8	10.3

**Table 4:** Foods consumed in the 24 h immediately preceding survey completion. Reported as percent of children consuming the food within the age group.

Food Categories	12 Months (*n* = 46)	24 Months (*n* = 46)	36 Months (*n* = 27)	*p*-Value
Cow Milk, %	60.9	91.3	81.5	0.002
Other Milk, %	6.5	13.0	0.0	0.119
Other Dairy, %	80.4	91.3	100	0.029
Other Soy, %	0.0	2.2	0.0	0.449
Baby Cereal, %	19.6	2.2	14.8	0.029
Other Grains, %	95.7	100	88.9	0.073
Meat, %	73.9	82.6	88.9	0.269
Liver, %	2.2	2.2	0.0	0.742
Beans/Lentils, %	21.7	28.3	22.2	0.734
Fruits, %	95.7	84.8	81.5	0.742
Vegetables, %	86.9	71.7	74.1	0.177
Carrots, %	47.8	26.1	51.9	0.039
Spinach, %	21.7	17.4	29.6	0.474
French Fries, %	13.0	21.7	26.0	0.352
100% Juice, %	19.6	54.3	55.6	<0.001
Sweet Food, %	26.1	50.0	59.3	0.010
Sweet Drinks, %	0.0	10.9	22.2	0.006
Vitamin D, %	10.8	4.3	3.7	0.356
Multivitamin, %	2.6	15.2	37.0	<0.001
Fish Oil, %	2.2	0	7.4	0.147
Other Supplements, %	4.3	4.3	11.1	0.422

**Table 5:** Foods that children had consumed at least once in the past year.

Food Categories	12 Months (*n* = 43)	24 Months (*n* = 45)	36 Months (*n* = 32)	*p*-Value
Comfort/Gentle Infant Formula, %	52.3	11.1	0.0	<0.001
Hypoallergenic Infant Formula, %	2.3	4.4	0.0	0.467
Human Milk, %	93.2	35.6	6.3	<0.001
Cowmilk Infant Formula, %	36.4	4.4	6.3	<0.001
Reflux Infant Formula, %	6.8	0.0	0.0	0.064
Soy Infant Formula, %	6.8	0.0	0.0	0.064
Other Infant Formula, %	11.4	2.4	0.0	0.041
Fruits, %	100	100	100	0.294
Vegetables, %	100	95.6	100	0.184
Barley, %	40.9	22.2	18.8	0.083
Oats, %	97.7	91.1	87.5	0.230
Animal Meat, %	93.2	100	100	0.064
Fruit Juice, %	52.3	84.4	87.5	0.230
Soda, %	6.8	24.4	31.3	0.022
Chocolate, %	56.8	86.7	100	<0.001
Candy, %	20.5	73.3	90.6	<0.001

**Table 6:** Results of statistical comparisons in alpha and beta diversity by diet diversity score.

Statistical Test	*p*-Value (Continuous Diet Diversity Score)	*p*-Value (8 Participants with 7 Groups vs. All Others)
Alpha Diversity (Kruskal–Wallis)		
Chao	0.2639	0.1615
Shannon	0.5655	0.5954
Inverse Simpson	0.5646	0.2882
Beta Diversity (Sorenson)		
Permanova	0.7221	0.3524
Permadisp	0.5730	0.4517
Beta Diversity (Bray–Curtis)		
Permanova	0.2245	0.1039
Permadisp	0.7481	0.3956

## Data Availability

The data presented in this study are available on request from the corresponding author on reasonable request.
